# LaVO_4_/Bi_2_O_2_S S-Scheme Heterojunction Driving Highly Efficient Photocatalytic Degradation of Tetracycline Antibiotics

**DOI:** 10.3390/molecules31193547

**Published:** 2026-10-05

**Authors:** Dongdong Chen, Yuhao Zeng, Yang Zhang, Fengli Cai, Chihpeng Lin, Bo Zhang, Shasha Liu, Zhenzhen Jia, Xiang Li

**Affiliations:** School of Environmental and Chemical Engineering, Zhaoqing University, Zhaoqing 526061, Chinazhangyang@zqu.edu.cn (Y.Z.); caifengli6416@126.com (F.C.); ftirlin@foxmail.com (C.L.); david_zhang200309@163.com (B.Z.); liushasha@zqu.edu.cn (S.L.); lixiang@zqu.edu.cn (X.L.)

**Keywords:** photocatalysis, TC degradation, high efficiency, S-scheme heterojunction, charge separation

## Abstract

The rational design and fabrication of semiconductor photocatalysts possessing high intrinsic activity are pivotal for the remediation of tetracycline (TC)-contaminated aqueous streams. Herein, we synthesized a LaVO_4_/Bi_2_O_2_S heterostructure and comprehensively interrogated its physicochemical attributes through a synergistic suite of techniques—X-ray diffraction (XRD), N_2_ adsorption/desorption isotherms, Fourier-transform infrared spectroscopy (FT-IR), UV–Vis diffuse reflectance spectroscopy (UV-Vis DRS), field-emission scanning and transmission electron microscopy (FE-SEM/TEM), X-ray photoelectron spectroscopy (XPS), steady-state photoluminescence (PL), and photoelectrochemical measurements. These analyses collectively revealed that the LVO/BOS composite with an equal mass ratio of LaVO_4_ and Bi_2_O_2_S manifested pronounced visible-light harvesting, markedly suppressed charge-carrier recombination, and accelerated interfacial charge transfer, as evidenced by enhanced photocurrent response and reduced PL intensity relative to the constituent LaVO_4_ and Bi_2_O_2_S phases. Consequently, under simulated solar irradiation, LVO/BOS achieved a TC degradation efficiency of ≈89% within 90 min, outperforming bare LaVO_4_ (41%) and Bi_2_O_2_S (44%). The corresponding apparent first-order rate constant (k ≈ 0.0214 min^−1^) exceeded those of LaVO_4_ (0.0031 min^−1^) and Bi_2_O_2_S (0.0053 min^−1^) by factors of 6.90 and 4.04, respectively. Radical-scavenging assays coupled with electron-spin-resonance (ESR) spectroscopy identified superoxide (•O_2_^−^) as the dominant reactive oxygen species driving TC mineralization. Guided by the experimentally determined band alignments and the observed preferential preservation of high-potential holes in LaVO_4_ and high-energy electrons in Bi_2_O_2_S, an S-scheme (step-scheme) heterojunction mechanism was postulated to rationalize the superior photocatalytic performance. This work underscores the strategic merit of S-scheme LaVO_4_/Bi_2_O_2_S heterostructures as robust, visible-light-driven platforms for the abatement of refractory organic pollutants.

## 1. Introduction

In recent years, water contamination has become a pressing global environmental challenge, threatening aquatic ecosystem integrity and water resource security across multiple societies. Discharges of harmful organic contaminants, particularly antibiotics, pose a grave threat to freshwater systems [1,2,3,4]. TC, a widely administered antibiotic, has emerged as a critical environmental pollutant due to its persistence, bioaccumulation potential, and role in propagating antibiotic-resistant genes [5,6]. To address this issue, a suite of conventional physical, biological, and chemical water treatment processes—including membrane filtration [7], adsorption [8], coagulation–flocculation [9] and biological treatment [10]—have been deployed to remove organic pollutants from water. Regrettably, these conventional processes fail to achieve effective mineralization of non-biodegradable substances, and are accompanied by considerable energy consumption and operational costs. In contrast, advanced oxidation technologies, especially semiconductor-based photocatalysis, have emerged as a highly promising solution: leveraging solar energy as a driving force, this technology can degrade TC in wastewater with high efficiency, cost-effectiveness, and environmental sustainability [1,2,11,12].

Vanadate-based semiconductors have emerged as a preeminent class of photocatalysts for organic pollutant degradation, owing to their tunable bandgaps and optimal band edge positions that align with the redox potentials of target degradation reactions [5,12,13,14]. Among them, lanthanum vanadate (LaVO_4_) has attracted extensive research interest as a high-potential visible-light-driven photocatalyst, attributed to its unique electronic and structural attributes: its valence band is dominated by V 3d orbitals, enabling efficient visible-light absorption via direct bandgap transitions, and it readily crystallizes into a thermodynamically stable monoclinic phase with high coordination symmetry, endowing it with robust structural stability under photocatalytic reaction conditions [13,15]. In recent years, monoclinic LaVO_4_ (*m*-LaVO_4_) has gained surging research attention, driven by its suite of desirable physicochemical and optoelectronic properties: a narrow bandgap of ~2.0 eV that overlaps well with the visible-light spectrum, low raw material cost, high visible-light responsivity, distinct monoclinic crystal architecture, and superior intrinsic catalytic activity [15,16,17]. Notwithstanding these merits, pristine LaVO_4_ suffers from intrinsic drawbacks that severely constrain its photocatalytic performance and large-scale practical deployment [13,15,18]. These limitations include a low specific surface area, insufficient accessible surface active sites, suboptimal visible-light harvesting capacity, and ultrafast recombination of photogenerated electron–hole (e^−^/h^+^) pairs, all of which collectively impede its photocatalytic efficiency and commercial translation.

To address these bottlenecks, a range of modification strategies have been explored, including element doping, morphology and structural engineering, and heterojunction construction [14,15,19]. Among these approaches, constructing heterojunctions with other semiconductor photocatalysts has been proven to be the most effective strategy to boost photocatalytic performance. Such heterostructures enable efficient spatial separation of photogenerated charge carriers, accelerated interfacial charge transfer kinetics, and broadened spectral light absorption range, collectively delivering photocatalytic performance far superior to that of single-component photocatalysts [1,5,15,19]. Particularly, ultrathin two-dimensional (2D) bismuth oxysulfide (Bi_2_O_2_S) has emerged as a highly promising coupling candidate for heterojunction construction, owing to its unique layered crystal structure and favorable optoelectronic properties. It crystallizes in a characteristic alternating stacking architecture of positively charged [Bi_2_O_2_]_2n_^2n+^ layers and S^2−^ anionic layers, held together by weak van der Waals interlayer interactions [20,21]. The intercalation of S^2−^ anions between the bismuth–oxygen cationic layers triggers synergistic structural and electronic modulation: it fine-tunes the band structure of the material, creates efficient interlayer charge transport pathways, and enhances the structural stability of the 2D framework under reaction conditions [20,21]. With a narrow bandgap ranging from 1.2 to 1.5 eV, Bi_2_O_2_S can efficiently harvest both visible and near-infrared (NIR) photons, substantially expanding its spectral response window to cover a broader fraction of the solar spectrum [20,21]. Additionally, Bi_2_O_2_S is composed of earth-abundant, low-toxicity elements, and can be synthesized via simple, scalable protocols, making it an ideal building block for sustainable photocatalytic systems [22]. Driven by these advantages, Bi_2_O_2_S has been widely employed as a functional component to construct heterojunctions with diverse semiconductor photocatalysts over the past two decades, leading to significantly boosted photocatalytic performance of the resulting composite systems. Representative heterojunctions reported to date include Bi_2_O_2_S/W_18_O_49_ [23], BiVO_4_/Bi_2_O_2_S [5], Bi_2_O_2_S/NiTiO_3_ [24], and Bi_2_O_2_S/In_2_O_3_ [25]. To the best of our knowledge, however, no prior studies have reported on the rational construction of LaVO_4_/Bi_2_O_2_S heterojunctions for photocatalytic applications, leaving a significant research gap in the combinatorial utilization of these two high-potential semiconductors with complementary optoelectronic properties.

Motivated by this research gap, in this work we report the facile hydrothermal synthesis of LaVO_4_/Bi_2_O_2_S heterojunctions for highly efficient visible-light-driven photocatalytic degradation of organic pollutants. The photocatalytic performance of the as-prepared samples was systematically evaluated via degradation of TC, a recalcitrant antibiotic pollutant ubiquitously detected in aquatic environments, as a model target contaminant. A comprehensive suite of characterization techniques, including structural, morphological, optical, and photoelectrochemical analyses, were employed to elucidate the chemical composition, micromorphology, and optoelectronic properties of the as-fabricated composites. Based on the systematic experimental results, a plausible S-scheme charge transfer pathway and corresponding photocatalytic degradation mechanism for the LaVO_4_/Bi_2_O_2_S heterostructure were proposed and discussed.

## 2. Results and Discussions

### 2.1. Catalysts Characterization

**Figure 1:**
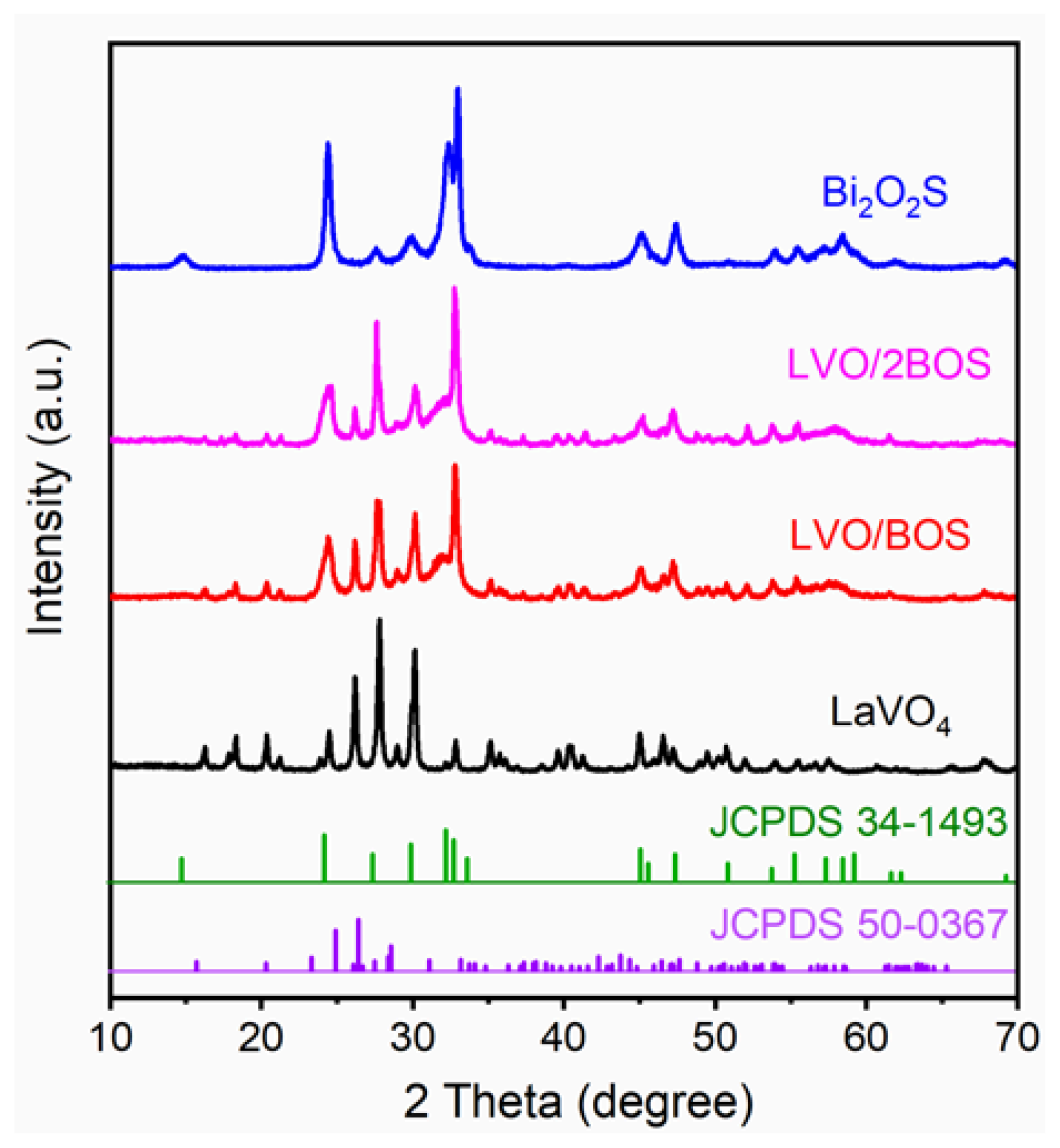
XRD patterns of as-obtained photocatalysts of LaVO_4_, Bi_2_O_2_S, LVO/BOS and LVO/2BOS composites.

The crystalline phase constitution and structural integrity of the pristine LaVO_4_, bare Bi_2_O_2_S, and LaVO_4_/Bi_2_O_2_S binary composites were first probed via XRD analysis. As presented in Figure 1, the XRD patterns of all three as-synthesized samples were systematically collected. Well-resolved diffraction peaks are discernible at 2θ = 24.46°, 26.18°, 27.79°, and 30.11°, which are unambiguously indexed to the (020), (200), (120) and (012) crystallographic planes of the well-crystallized *m*-LaVO_4_ phase (JCPDS No. 50-0367) [15,17,26]. For the bare Bi_2_O_2_S reference sample, its XRD pattern exhibits a set of distinct peaks at 2θ = 24.23°, 29.96°, 32.29°, 32.78°, 45.04°, and 47.36°, which are consistently assigned to the (110), (040), (130), (101), (141), and (002) planes of the layered orthorhombic Bi_2_O_2_S phase (JCPDS No. 34-1493) [5,27]. For the LaVO_4_/Bi_2_O_2_S composites, their XRD patterns concurrently manifest all fingerprint diffraction peaks inherent to both constituent LaVO_4_ and Bi_2_O_2_S phases, directly verifying the successful in-situ assembly of the two-phase heterostructures without observable phase separation. Notably, no extraneous impurity peaks are detected across the full 2θ scanning range of any composite pattern, confirming the high phase purity of the as-fabricated materials. Moreover, a clear compositional dependence of the diffraction peak intensities is observed: with decreasing LaVO_4_ loading content in the composites, the relative intensities of the Bi_2_O_2_S characteristic peaks increase monotonically, which further corroborates the successful fabrication of the targeted LaVO_4_/Bi_2_O_2_S heterostructures.

**Figure 2:**
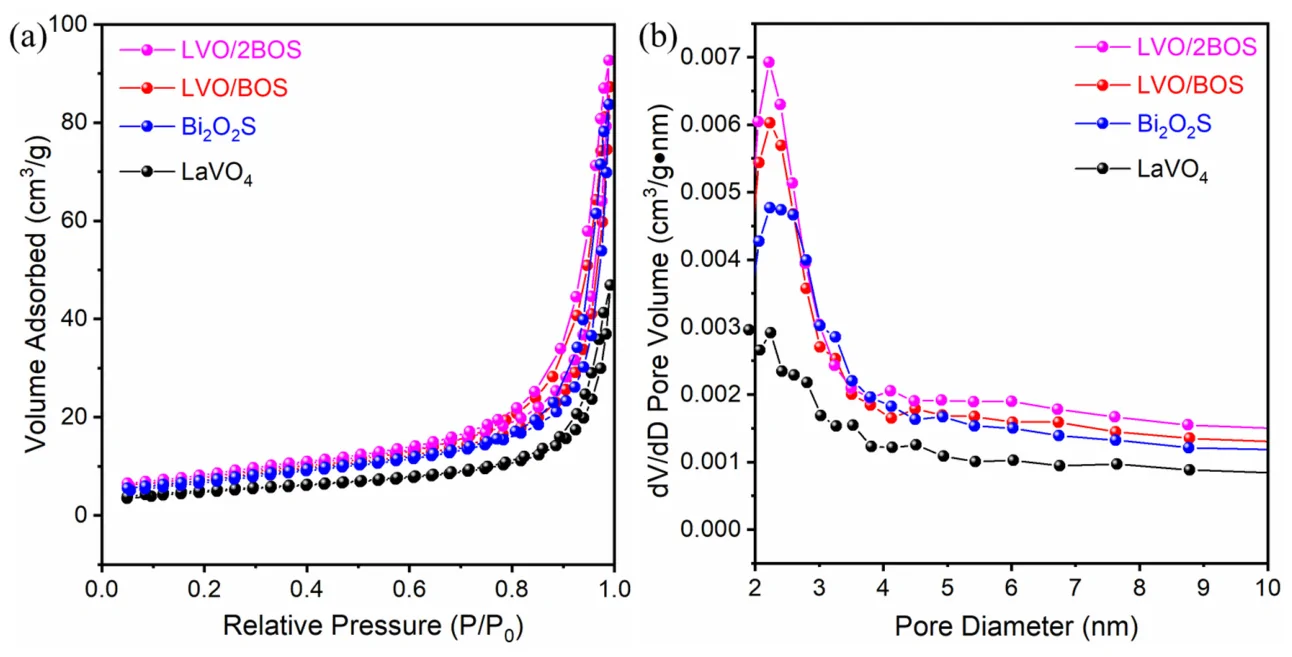
(**a**) N_2_ adsorption–desorption curves and (**b**) the corresponding average pore size distribution plots of LaVO_4_, Bi_2_O_2_S, LVO/BOS and LVO/2BOS composites.

The specific surface area and pore structural characteristics of the as-prepared photocatalysts were systematically characterized via N_2_ adsorption–desorption isotherms at 77 K, with the corresponding data summarized in Figure 2. As depicted in Figure 2a, all the as-synthesized samples present typical Type-IV adsorption isotherms accompanied by H3-type hysteresis loops, a signature feature of mesoporous materials reported in the literature [5]. The specific surface area (S_BET_) of each sample was calculated in strict accordance with the Brunauer–Emmett–Teller (BET) algorithm. The calculated S_BET_ values for pristine LaVO_4_, Bi_2_O_2_S, LVO/BOS and LVO/2BOS heterojunctions are 17.0, 24.5, 26.6 and 28.9 m^2^/g, respectively. These results evidently demonstrate that the introduction of Bi_2_O_2_S induces a marked augmentation in the specific surface area of the hybrid photocatalysts, relative to the single-phase LaVO_4_. The pore size distribution curves of the as-prepared samples were derived from the Barrett–Joyner–Halenda (BJH) model based on the desorption branch, as presented in Figure 2b. The average pore diameters of the LaVO_4_, Bi_2_O_2_S, LVO/BOS and LVO/2BOS are 15.7, 21.0, 19.5 and 19.8 nm, respectively, while the corresponding pore volumes are 0.07, 0.13, 0.13 and 0.14 cm^3^/g, respectively. These results further corroborate the successful construction of a hierarchical mesoporous architecture within the LaVO_4_/Bi_2_O_2_S heterostructure. This unique structural feature endows the LaVO_4_/Bi_2_O_2_S composites with abundant accessible active sites for TC adsorption and subsequent photocatalytic degradation, thereby positioning them as highly promising candidates for high-efficiency photocatalytic applications [28].

**Figure 3:**
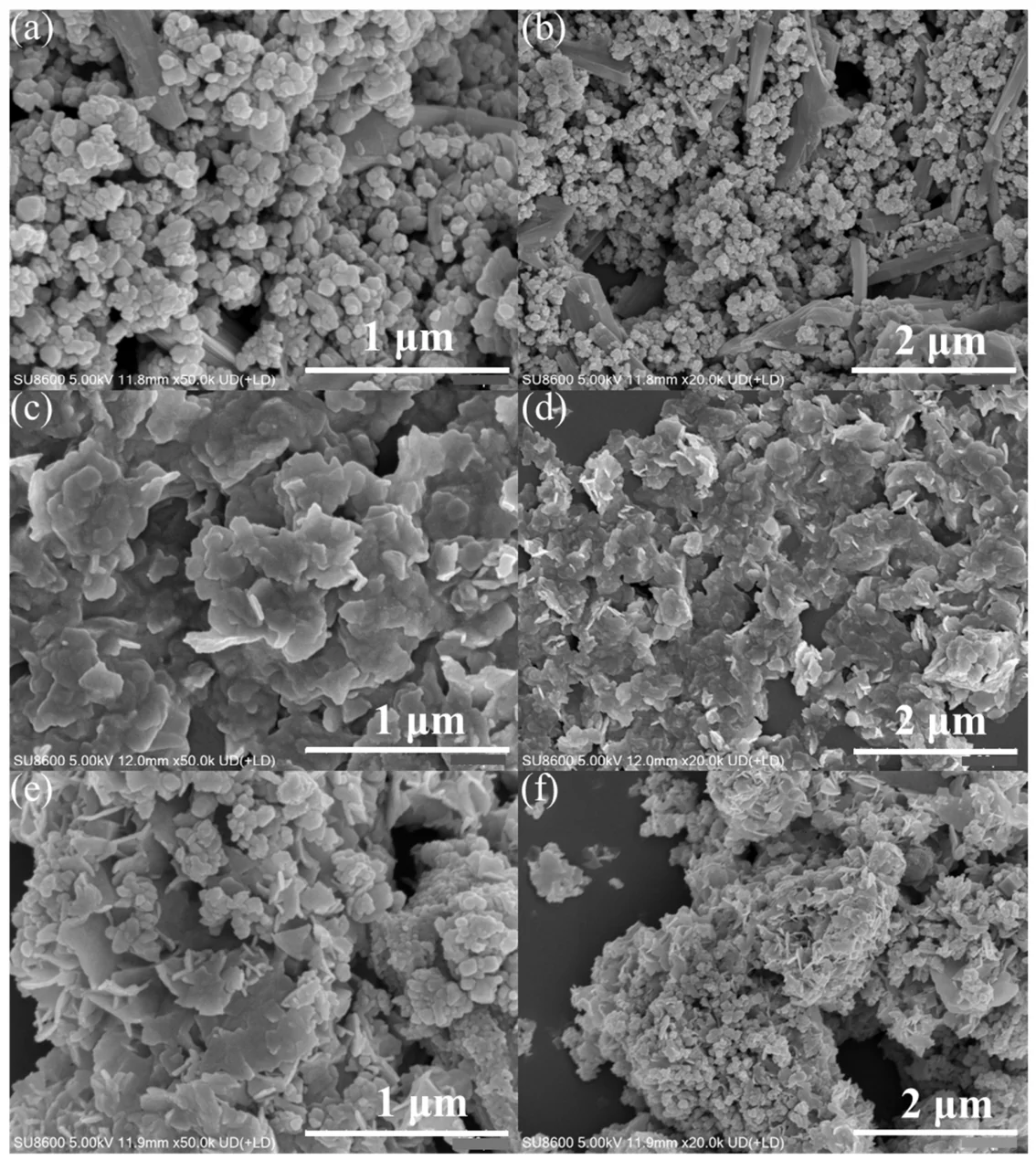
Representative FE-SEM images of LaVO_4_ (**a**,**b**), Bi_2_O_2_S (**c**,**d**), and LVO/BOS composite (**e**,**f**) at different magnifications.

**Figure 4:**
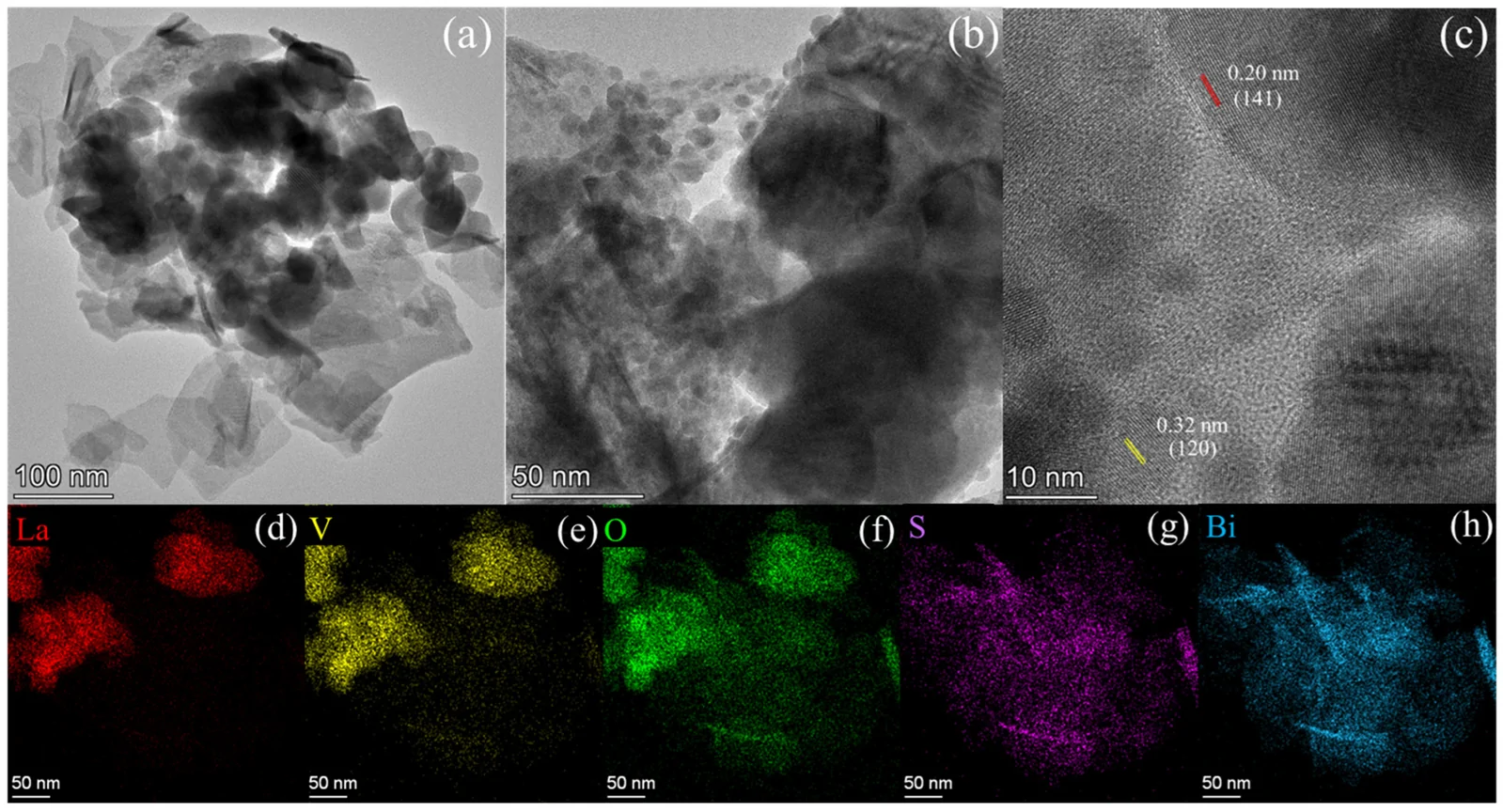
TEM (**a**,**b**), HRTEM images (**c**), and the EDX elemental mapping images (**d**–**h**) of LVO/BOS composite (La, V, O, S and Bi elements). Note: Red and yellow lines in panel (**c**) denote Bi_2_O_2_S and LaVO_4,_ respectively.

The morphology of the as-synthesized specimens was first interrogated by FE-SEM and TEM. As illustrated in Figure 3a,b, pristine LaVO_4_ manifests as a dense agglomeration of irregular, nanoscale particles. Conversely, Figure 3c,d disclose that Bi_2_O_2_S adopts a distinctly lamellar architecture—a structural motif that is wholly congruent with the observations reported in our earlier investigation [5]. In the LVO/BOS heterojunction composite (Figure 3e,f), the disparate morphologies of the irregular LaVO_4_ nanoparticles and the ultrathin Bi_2_O_2_S nanosheets are readily discernible, thereby attesting to the simultaneous presence of both phases within the hybrid. To procure deeper structural insight, complementary TEM and high-resolution TEM (HRTEM) analyses were undertaken (Figure 4). Notably, the HRTEM images revealed a profusion of LaVO_4_ nanoparticles uniformly dispersed upon the surface of Bi_2_O_2_S nanosheets, evidencing successful hybridization. This intimate interfacial contact engendered a synergistic electronic coupling between the LaVO_4_ and Bi_2_O_2_S constituents, which was conducive to enhanced charge-carrier separation and transfer. The HRTEM micrograph displayed in Figure 4c exhibited unambiguous lattice fringes at 0.20 nm, assignable to the (141) plane of Bi_2_O_2_S, and at 0.32 nm, correlating with the (120) plane of LaVO_4_, furnishing definitive proof of the LaVO_4_/Bi_2_O_2_S heterojunction formation. Complementary energy-dispersive X-ray spectroscopy (EDS) elemental mapping (Figure 4d–h) further substantiated the homogeneous spatial distribution of La, V, O, Bi, and S throughout the LVO/BOS architecture, each element rendered in a distinct chromatic code. It could be observed from the elemental mapping that La and V exhibit identical overlapping regions, while Bi and S showed the same co-localization pattern. Moreover, the oxygen mapping revealed both of these overlapping areas, confirming the presence of LaVO_4_ and Bi_2_O_2_S and indicating that the two phases are closely integrated with each other. Collectively, these multimodal characterizations furnished compelling, robust evidence for the successful synthesis, structural fidelity, and compositional uniformity of the LVO/BOS photocatalyst, thereby establishing a solid platform for its pronounced photocatalytic performance.

**Figure 5:**
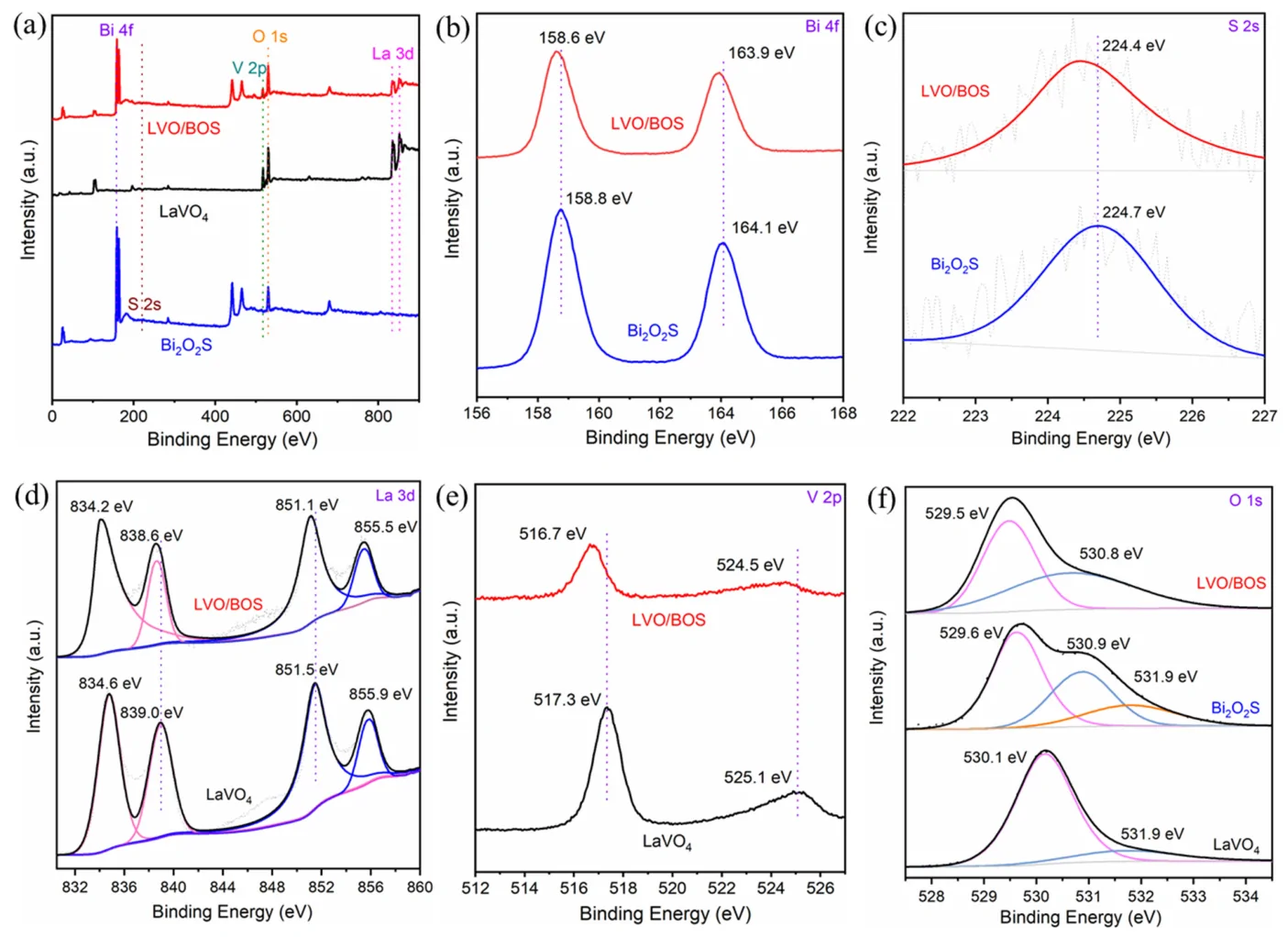
XPS spectra: (**a**) survey spectra, (**b**) Bi 4f, (**c**) S 2s, (**d**) La 3d, (**e**) V 2p and (**f**) O 1s for the photocatalysts.

To systematically decode the elemental constitution and surface chemical valence states of the as-synthesized photocatalysts, rigorous XPS characterizations were performed on bare LaVO_4_, monolithic Bi_2_O_2_S, and the engineered LVO/BOS heterojunction, with the corresponding analytical outcomes compiled and presented in Figure 5. As depicted in Figure 5a, the wide-scan survey spectra of bare LaVO_4_ and monolithic Bi_2_O_2_S exclusively display signature peaks assignable to La, V, O and Bi, O, S, respectively. In contrast, the survey spectrum of the LVO/BOS composite integrates all aforementioned elemental signatures, providing unequivocal evidence for the successful construction of the binary heterojunction. Subsequent deconvolution of high-resolution XPS fine spectra was carried out to interrogate the chemical valence states of each constituent element. For the high-resolution Bi 4f spectrum of bare Bi_2_O_2_S (Figure 5b), two deconvoluted peaks centered at 164.1 eV and 158.8 eV are unambiguously ascribed to Bi 4f_7/2_ and Bi 4f_5/2_, respectively. Notably, relative to the peaks observed in bare Bi_2_O_2_S, the Bi 4f peaks of the LVO/BOS heterojunction display a marked negative shift in binding energy, moving to 163.9 eV for Bi 4f_7/2_ and 158.6 eV for Bi 4f_5/2_. This negative shift phenomenon is extensively reported in previously documented heterojunction systems with analogous interfacial charge transfer characteristics, and can be attributed to interfacial charge redistribution driven by the superior intrinsic conductivity of Bi_2_O_2_S [5,29], thereby offering compelling evidence for robust interfacial interactions between LaVO_4_ and Bi_2_O_2_S. Furthermore, the spin–orbit splitting energy of Bi 4f remains invariant at 5.3 eV for both bare Bi_2_O_2_S and the LVO/BOS composite, confirming that Bi maintains a +3 oxidation state across the single-component materials and the heterojunction [21,29]. Additionally, the high-resolution S 2s spectra presented in Figure 5c indicate that S persists in a -2 oxidation state in both the bare Bi_2_O_2_S and LVO/BOS composite [21]. Figure 5d presents the deconvoluted results of the high-resolution La 3d spectra: as illustrated, four peaks are resolved for both samples, with peaks at 834.6 eV and 839.0 eV assigned to La 3d_3/2_, and peaks at 851.5 eV and 855.9 eV assigned to La 3d_5/2_ for bare LaVO_4_; for the LVO/BOS composite, the corresponding peaks shift slightly to 834.2 eV, 838.6 eV, 851.1 eV and 855.5 eV, respectively. The spin–orbit splitting energy of ~4.4 eV further confirms that La adopts a +3 valence state in both bare LaVO_4_ and the LVO/BOS composite [14,16,26]. Figure 5e displays the high-resolution V 2p spectrum acquired from bare LaVO_4_, wherein two signature V 2p peaks are resolved. Specifically, the peaks at 517.3 eV and 525.1 eV are assigned to V 2p_3/2_ and V 2p_1/2_, respectively [30]. In the LVO/BOS composite, these V 2p peaks shift to lower binding energies compared to pure LaVO_4_, a phenomenon attributable to strong electronic coupling between LaVO_4_ and Bi_2_O_2_S [30]. This observation can be ascribed to the presence of V in its pentavalent oxidation state (V^5+^ in VO_4_^3−^ units) in the LaVO_4_ samples, and the characteristic V^5+^ peak is also discernible in the LVO/BOS composite [16]. Figure 5f shows the high-resolution O 1s spectrum of the as-synthesized photocatalysts. For monolithic Bi_2_O_2_S, three resolved O 1s signals at binding energies of 529.6 eV, 530.9 eV and 531.9 eV are detected, which are ascribed to lattice oxygen associated with Bi–O bonds, oxygen vacancies, and surface hydroxyl groups, respectively [22,31]. While the bare LaVO_4_ exhibits only two distinct O 1s peaks located at 530.1 eV and 531.9 eV, corresponding to lattice oxygen and oxygen vacancies, respectively [26,32]. Notably, the O 1s peaks in the LVO/BOS composite exhibit a slight negative shift in binding energy relative to the single-component precursors, further providing compelling evidence for the formation of robust interfacial electronic interactions between LaVO_4_ and Bi_2_O_2_S.

**Figure 6:**
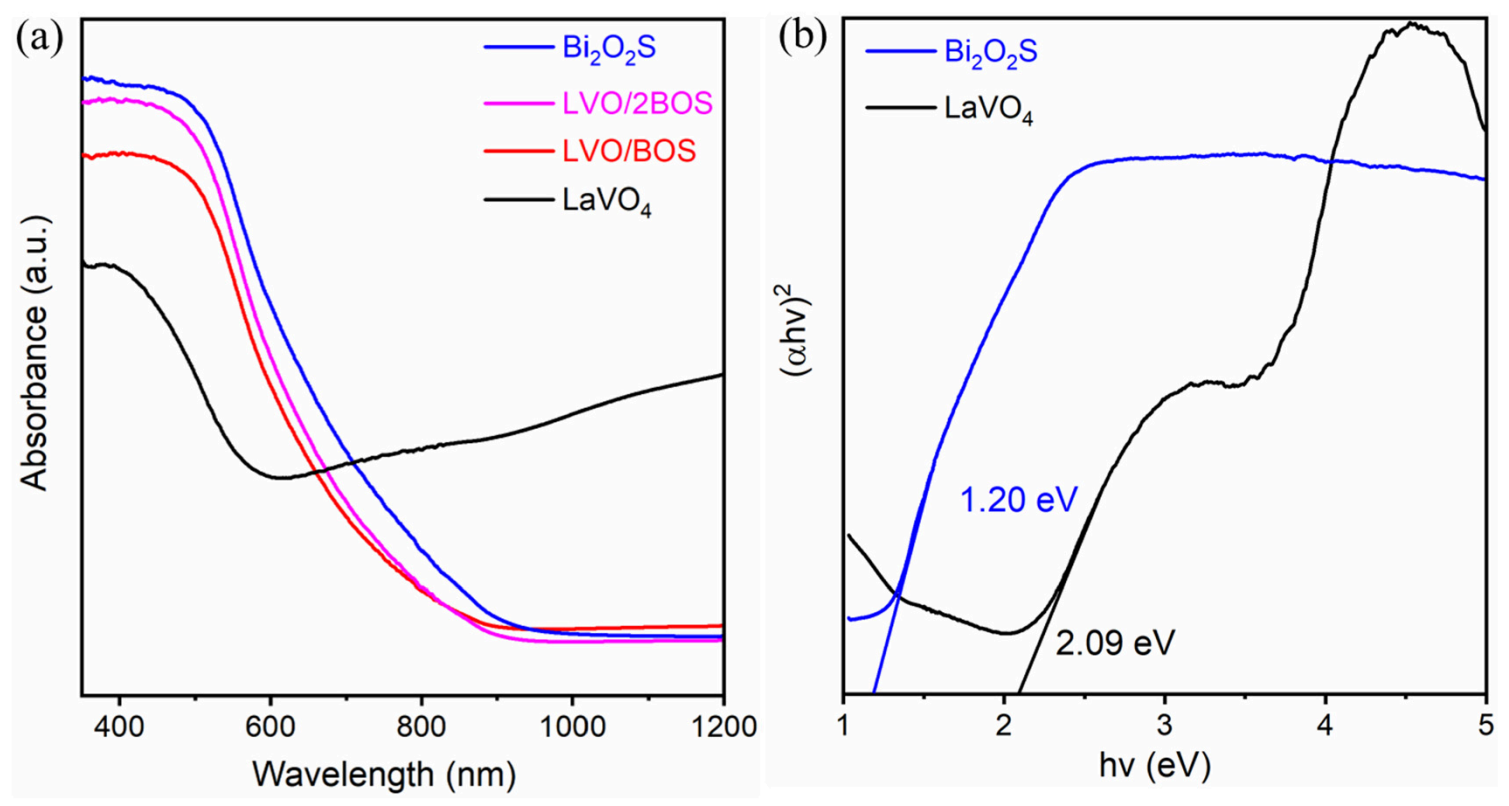
(**a**) UV-Vis spectra of LaVO_4_, Bi_2_O_2_S, LVO/BOS and LVO/2BOS composite and (**b**) corresponding Tauc plots for LaVO_4_ and Bi_2_O_2_S.

The spectral light-harvesting capability of a semiconductor serves as a pivotal determinant governing its photocatalytic activity [5,33,34,35]. To this end, UV-Vis DRS characterization was systematically conducted on the as-synthesized photocatalysts to elucidate their optical response profiles and derive fundamental electronic structure parameters. As evident from the UV-Vis DRS profiles in Figure 6a, pristine LaVO_4_ exhibits light absorption confined to a narrow window of the visible spectrum. In contrast, the absorption band edge of the LaVO_4_/Bi_2_O_2_S heterojunction composite undergoes a distinct redshift relative to pure LaVO_4_, signifying an extended photo-response range. This result confirms that the incorporation of Bi_2_O_2_S substantially enhances the visible-light harvesting capacity of LaVO_4_, far surpassing the performance of the single-phase material. Furthermore, a composition-dependent shift in the absorption edge is discernible: as the Bi_2_O_2_S loading increases, the absorption edge of the composite progressively migrates toward that of pristine Bi_2_O_2_S, verifying that heterojunction construction effectively modulates the optical properties of the LaVO_4_ matrix. To further elucidate the electronic structure of the samples, the bandgap energy (Eg) of the semiconductors was derived from the Kubelka–Munk (K-M) function, following the established empirical relationship: αhν = A(hν − Eg)^n^, where α denotes the absorption coefficient, h represents Planck’s constant, ν refers to the incident photon frequency, A is an empirical constant, and the exponent n is determined by the nature of the electronic transition [5,34,35]. Based on the Tauc plots (Figure 6b) constructed from the UV-Vis DRS data, the bandgap energies of pristine LaVO_4_ and Bi_2_O_2_S are determined to be approximately 1.20 eV and 2.09 eV, respectively.

**Figure 7:**
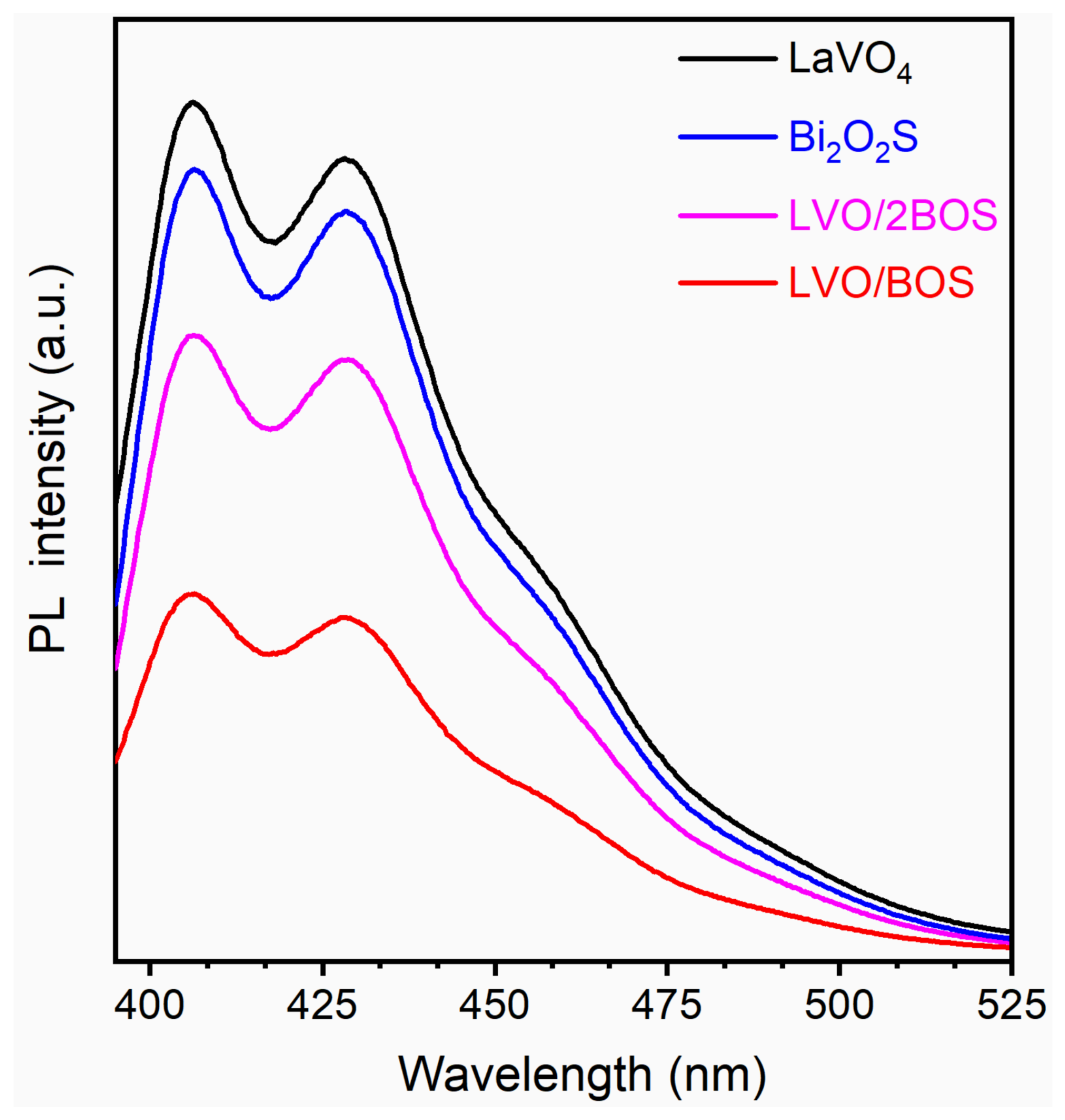
PL spectra of LaVO_4_, Bi_2_O_2_S, LVO/BOS and LVO/2BOS composites.

**Figure 8:**
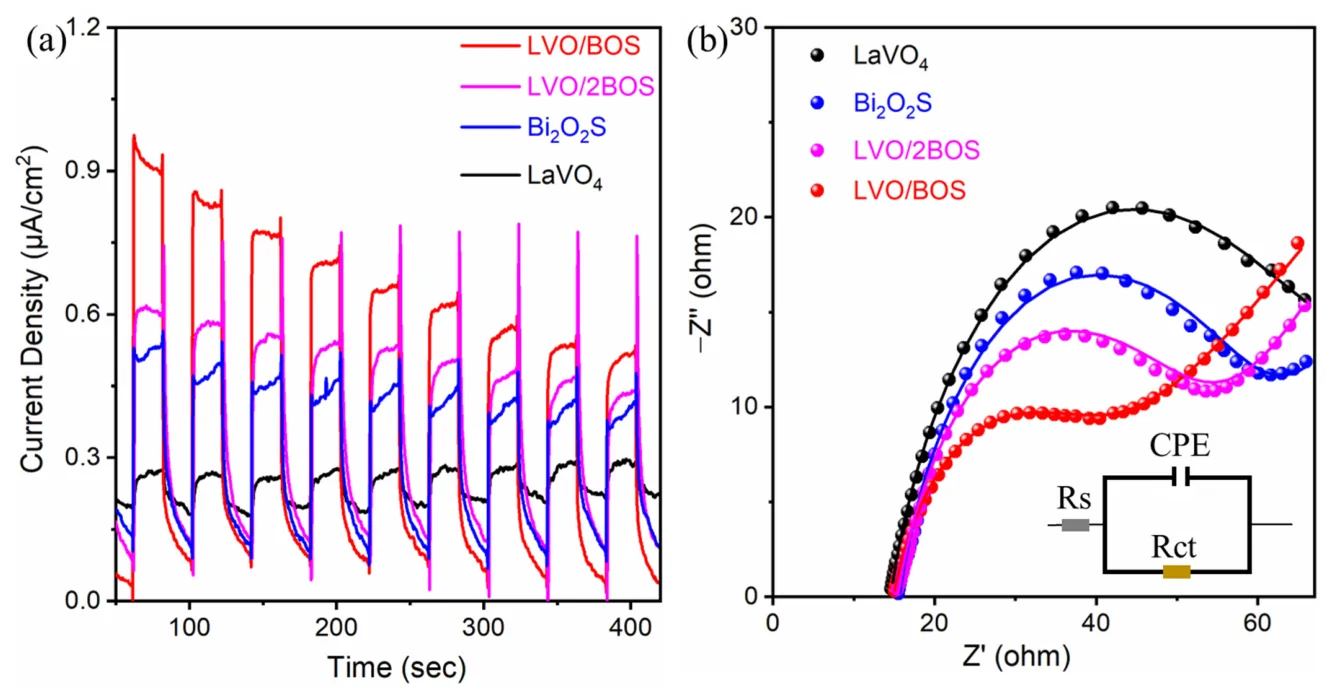
(**a**) Transient photocurrent response and (**b**) EIS of LaVO_4_, Bi_2_O_2_S, LVO/BOS and LVO/2BOS composites.

Steady-state PL spectroscopy was first deployed to interrogate the intrinsic optoelectronic properties of the as-synthesized photocatalysts, specifically quantifying the separation efficiency, recombination kinetics, and interfacial charge transfer dynamics of photogenerated carriers. As a well-established characterization benchmark in photocatalysis, the PL emission intensity exhibits an inherent inverse correlation with the separation efficiency of photogenerated e^−^-h^+^ pairs: diminished PL intensity signifies suppressed radiative recombination of charge carriers, which in turn elevates the probability of effective charge migration to reactive sites, ultimately endowing the material with superior photocatalytic performance [5,15,26]. As depicted in Figure 7, the intense PL emission peaks observed for the pristine LaVO_4_ and Bi_2_O_2_S single-component semiconductors indicate severe charge carrier recombination within these materials, where the vast majority of photogenerated e^−^-h^+^ pairs recombine via radiative pathways before they can be extracted for catalytic reactions. By contrast, the LaVO_4_/Bi_2_O_2_S heterojunction composites exhibit markedly suppressed PL emission relative to both individual LaVO_4_ and Bi_2_O_2_S components, directly evidencing the enhanced separation efficiency of photogenerated e^−^-h^+^ pairs across the heterointerface. Among all prepared samples, the LVO/BOS composite delivers the most pronounced PL quenching effect, registering the lowest PL emission intensity, which correlates strongly with its optimal photocatalytic activity. To further validate the charge separation and migration behavior inferred from the PL results, complementary photoelectrochemical characterizations, including transient photocurrent response measurements and electrochemical impedance spectroscopy (EIS), were subsequently performed to quantify the dynamic transport kinetics of photogenerated carriers at the electrode/electrolyte interface [5,36,37]. As presented in Figure 8a, the LaVO_4_/Bi_2_O_2_S composites deliver substantially higher photocurrent densities than the individual LaVO_4_ and Bi_2_O_2_S electrodes, directly confirming the faster interfacial charge transfer kinetics across the heterojunction interface in the composite systems. Further mechanistic insights into the interfacial charge transfer processes were obtained via EIS analysis, with the corresponding Nyquist plots presented in Figure 8b. As a standard characterization for interfacial charge transport properties, the EIS spectra of all samples typically comprise three frequency-dependent components: (i) ohmic resistance (R_s_) originating from the electrolyte, electrode substrate, and external circuit connections; (ii) charge transfer resistance (Rct) representing the kinetic barrier for charge migration across the electrode/electrolyte interface; and (iii) a constant phase element (CPE) introduced to correct for the non-ideal capacitive behavior arising from surface roughness and heterogeneous charge distribution at the electrode surface [5,17,36,37]. It is widely acknowledged that a smaller Rct value corresponds to faster charge transfer kinetics at the electrode/electrolyte interface, and the magnitude of Rct can be directly visualized by the radius of the semicircular arc in the high-frequency region of the Nyquist plot [5,17,36,37]. As explicitly shown in Figure 8b and Table 1, the LVO/BOS heterostructure delivers the lowest charge-transfer resistance (Rct) among all evaluated samples. This result directly reflects its superior interfacial charge transfer capability relative to the other tested systems. This minimized interfacial resistance facilitates rapid migration of photogenerated carriers to the electrode surface for participation in redox reactions, thereby providing additional support for its superior photocatalytic performance.

### 2.2. Photocatalytic Degradation of TC

**Figure 9:**
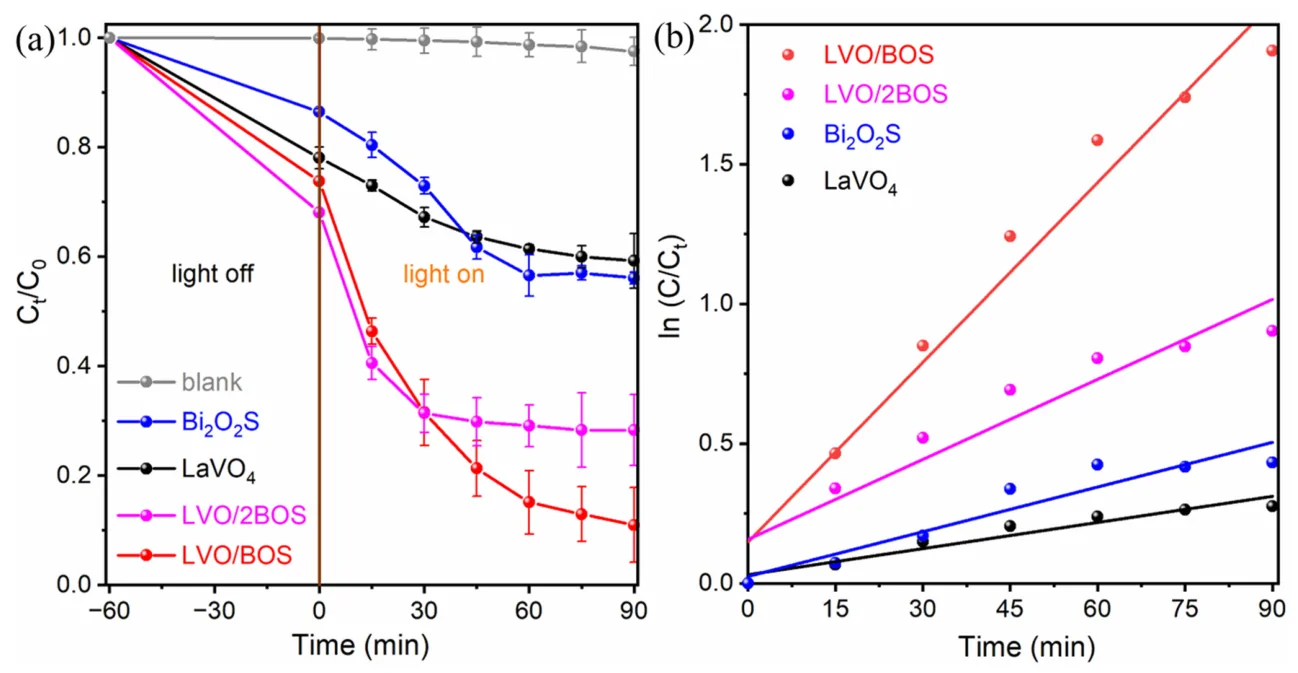
(**a**) Photocatalytic degradation of TC (C_0_ represents the initial TC concentration) and (**b**) pseudo-first-order reaction kinetics of the LaVO_4_, Bi_2_O_2_S, LVO/BOS, and LVO/2BOS heterostructure.

A recalcitrant antibiotic pollutant ubiquitously detected in aquatic ecosystems, was selected as a model target contaminant to benchmark the visible-light photocatalytic activity of the four as-fabricated catalysts [38]. Prior to light irradiation, a 60-min dark adsorption pre-treatment was conducted to establish adsorption–desorption equilibrium across the entire reaction system, eliminating the interference of adsorption-induced concentration fluctuations on subsequent photocatalytic degradation quantification. The visible-light photocatalytic degradation profiles of TC over the as-synthesized samples are compiled in Figure 9; notably, the blank control experiment of pristine TC self-degradation exhibited negligible conversion (<3% after 90 min irradiation), confirming that TC loss in the absence of catalysts can be safely excluded from subsequent performance evaluation. As illustrated in Figure 9a, the monolithic LaVO_4_ and Bi_2_O_2_S single-component catalysts demonstrated comparatively low photocatalytic degradation efficiencies of 41% and 44%, respectively, after 90 min of visible-light irradiation. This limited photocatalytic activity is primarily ascribed to the ultrafast recombination of photogenerated e^−^-h^+^ pairs within the single-component semiconductor lattices, which severely restricts the supply of active redox species for TC mineralization. In sharp contrast, the LVO/BOS and LVO/2BOS binary heterostructures demonstrated markedly superior photocatalytic performance, achieving TC degradation efficiencies of ~89% and 72% over the same 90 min irradiation period, far outperforming both individual LaVO_4_ and Bi_2_O_2_S components. This pronounced enhancement in photocatalytic activity of the LaVO_4_/Bi_2_O_2_S heterostructures can be rationalized by two synergistic factors: (i) the S-scheme charge transfer pathway at the LaVO_4_/Bi_2_O_2_S interface that effectively suppresses e^−^-h^+^ recombination and prolongs the lifetime of photogenerated charge carriers; (ii) the broadened visible-light absorption range and enhanced light-harvesting capacity enabled by the coupled semiconductor system, which generates a higher density of photogenerated carriers under visible illumination.

To further elucidate the intrinsic degradation kinetics of TC over the different catalysts, the photocatalytic degradation data were fitted to a pseudo-first-order kinetic model, which is widely adopted to describe photocatalytic degradation processes of organic pollutants under mild reaction conditions [5,38,39]. As displayed in Figure 9b, the apparent rate constants of the four catalysts were derived from linear regression of ln(C/C_t_) against irradiation time, where C and C_t_ represent the residual tetracycline concentration after dark adsorption and instantaneous TC concentration at reaction time t, respectively. Based on the kinetic fitting results, the apparent rate constants of the four catalysts were extracted quantitatively. As summarized in Figure 9b, the LVO/BOS heterostructure exhibits the maximum k value of 0.0214 min^−1^, which is significantly higher than those of the pristine LaVO_4_ (0.0031 min^−1^), Bi_2_O_2_S (0.0053 min^−1^), and LVO/2BOS (0.0095 min^−1^) catalysts. Notably, the k of the optimal LVO/BOS heterostructure is approximately 6.90-fold, 4.04-fold, and 2.25-fold higher than those of the pristine LaVO_4_, Bi_2_O_2_S, and LVO/2BOS catalysts, respectively. This marked amplification in photocatalytic activity provides compelling evidence for the synergistic effect generated by the rational integration of LaVO_4_ and Bi_2_O_2_S components into the binary heterostructure, further underscoring the excellent interfacial charge transfer dynamics and superior visible-light photocatalytic efficiency of the LaVO_4_/Bi_2_O_2_S composite system.

**Figure 10:**
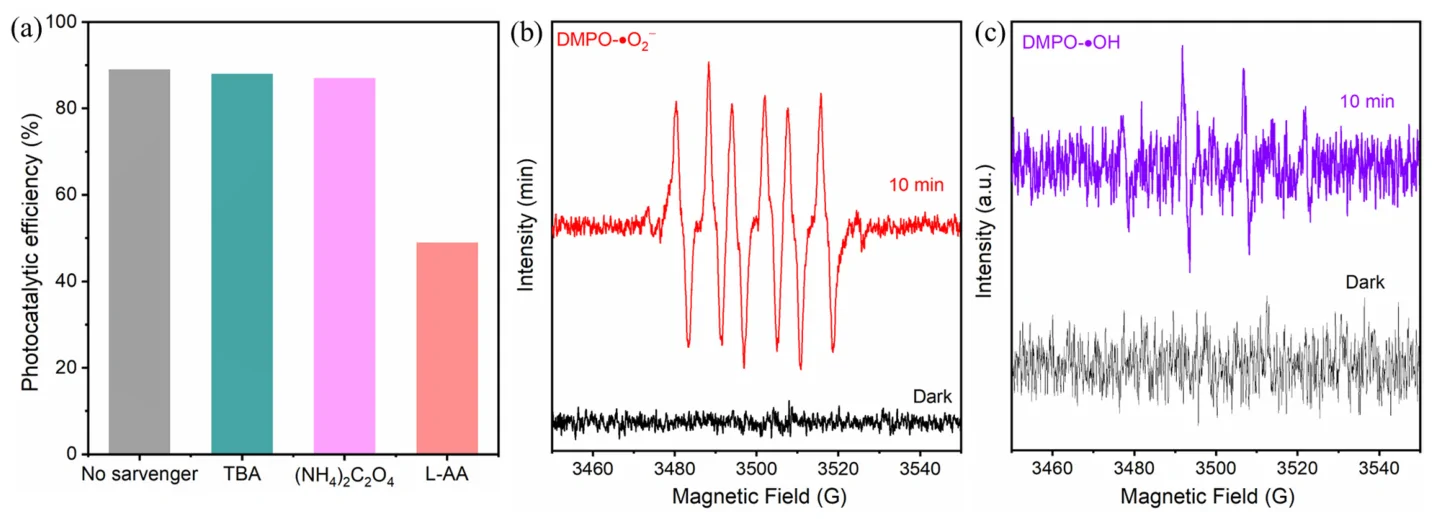
(**a**) Photocatalytic TC degradation performance of LVO/BOS heterostructure with various radical scavengers; ESR spectra of LVO/BOS for DMPO-•O_2_^−^ (**b**) and DMPO-•OH (**c**).

Reactive species scavenging experiments constitute a core methodology for deconvoluting the charge transfer pathway and reaction mechanism of heterogeneous photocatalysts. To further decipher the dominant reactive species orchestrating photocatalytic TC degradation over the LVO/BOS heterojunction, a rigorously designed series of species-specific quenching tests was systematically executed under uniform visible-light irradiation conditions, with strict control of all other reaction parameters to guarantee the statistical robustness and inter-group comparability of the results. In reactive species scavenging experiments, ammonium oxalate ((NH_4_)_2_C_2_O_4_), 10 mmol L^−1^), L-ascorbic acid (L-AA, 1 mmol L^−1^), and tert-butyl alcohol (TBA, 2 mL) were employed as targeted scavengers to selectively quench photogenerated h^+^, •O_2_^−^, and •OH, respectively [12,40]. As illustrated in Figure 10a, the blank control group (without any scavenger addition) exhibited a TC degradation efficiency of 89%, which was defined as the normalized reference for quantifying the individual contribution of each reactive species. Notably, the addition of L-AA induced a pronounced 40% suppression of TC degradation, with the removal efficiency plummeting to 45%—this dramatic inhibition unambiguously identifies •O_2_^−^ as the primary reactive species driving TC mineralization. In contrast, the introduction of (NH_4_)_2_C_2_O_4_ and TBA only triggered marginal suppressive effects, with degradation efficiencies of 87% and 88% recorded, corresponding to inhibition extents of ~2% and ~1%, respectively; these modest inhibitions confirm that h^+^ and •OH act as secondary reactive species with negligible contributions to the overall degradation process. To further substantiate the in-situ generation of reactive oxygen species during the photocatalytic degradation of TC over the LVO/BOS composite, ESR spectra were systematically acquired. As exhibited in Figure 10b,c, no discernible signals corresponding to the DMPO-•OH and DMPO-•O_2_^−^ adducts were detected under dark conditions, whereas distinct characteristic signals of the DMPO-•O_2_^−^ adduct emerged within a 10-min visible-light irradiation period, accompanied by faint but discernible signals of the DMPO-•OH adduct. These observations confirm the light-driven generation of both •OH and •O_2_^−^ radicals on the LVO/BOS surface under visible-light illumination. Notably, •O_2_^−^ radicals constitute the predominant reactive species in this system, while •OH radicals play a comparatively subordinate role, a conclusion that aligns perfectly with the outcomes of radical trapping experiments.

**Figure 11:**
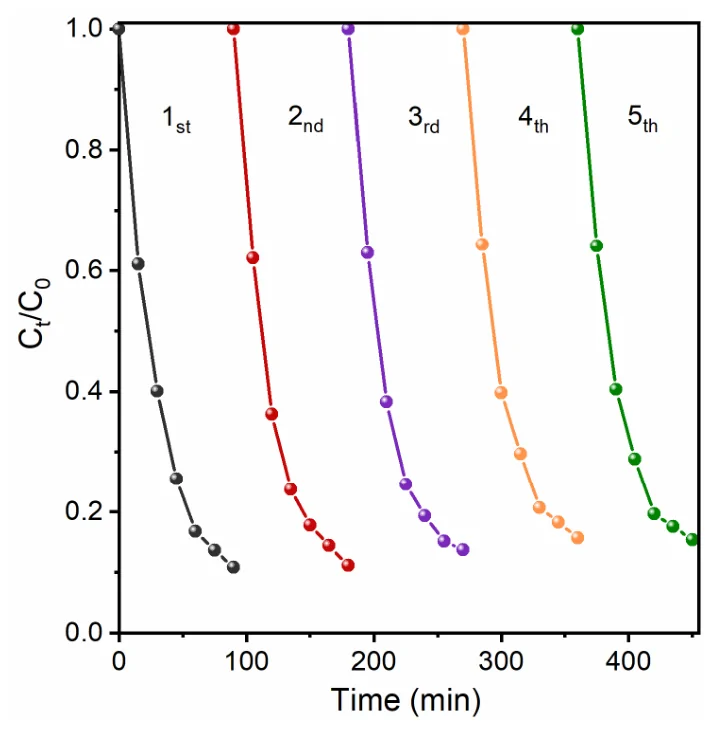
Five-cycle recycling test of LVO/BOS heterostructure for photocatalytic TC degradation.

Stability constitutes a cardinal metric for gauging the translational viability of high-performance photocatalysts, especially under the stringent operational demands of refractory wastewater decontamination. Consequently, a systematic and rigorous evaluation of the operational reusability and long-term structural integrity of the LVO/BOS heterostructure is of paramount importance for its practical deployment. To quantitatively probe its cyclic durability, five consecutive photocatalytic degradation runs of TC under simulated visible-light irradiation were performed, with the resultant degradation efficiencies summarized in Figure 11. Encouragingly, relative to the initial catalytic cycle, the heterostructure displayed a negligible attenuation of merely ~4% in TC degradation efficiency after five successive runs. This minimal performance decay can be rationally ascribed to minor material abrasion or partial shedding of surface active sites, both of which are triggered by iterative light irradiation and subsequent catalyst recovery procedures.

### 2.3. Photocatalytic Mechanism

**Figure 12:**
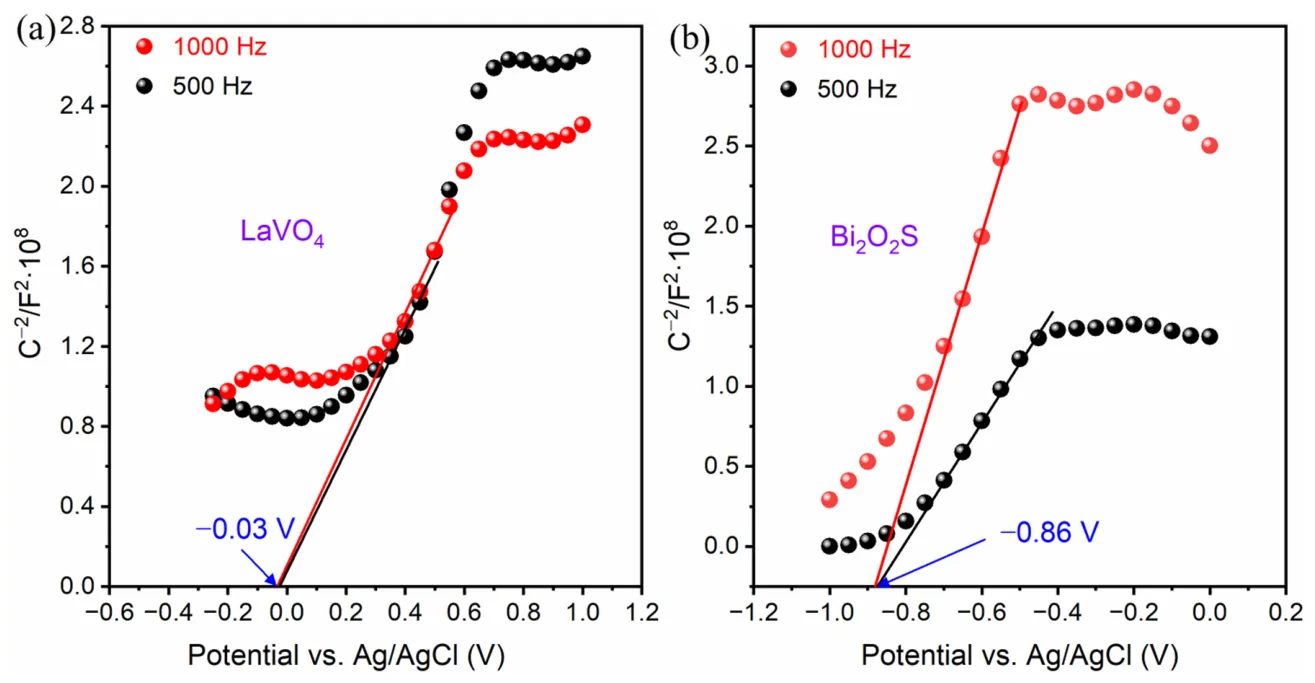
Mott−Schottky plots for different catalysts at specific frequencies (1000 and 500 Hz) obtained in darkness, (**a**) LaVO_4_; (**b**) Bi_2_O_2_S.

To construct a rigorous energy band framework for rational interpretation of the photocatalytic behavior of the LVO/BOS composite, Mott–Schottky (M-S) electrochemical profiling was performed to precisely quantify the flat-band potential (E_fb_) of each semiconductor component. Cross-referenced with UV-Vis DRS-derived Eg values, this dataset enables the quantitative derivation of the absolute conduction band minimum (E_CB_) and valence band maximum (E_VB_) positions for both phases. As depicted in Figure 12, the M–S plots of both LaVO_4_ and Bi_2_O_2_S exhibit typical positive slopes across the measured potential window, an intrinsic electrochemical signature of n-type semiconductors, thereby directly confirming the n-type semiconducting nature of the two constituents. The E_fb_ values were extrapolated from the x-intercepts of the linear regimes of the M–S plots referenced to the Ag/AgCl electrode, yielding values of −0.03 V for LaVO_4_ and −0.86 V for Bi_2_O_2_S, respectively. Under the tested pH = 7 condition, these Ag/AgCl-referenced potentials were converted to the normal hydrogen electrode (NHE) scale via the Nernst equation (E_NHE_ = E_Ag/AgCl_ + 0.197, pH = 7), affording calibrated E_fb_ values of 0.17 V and −0.66 V for LaVO_4_ and Bi_2_O_2_S, respectively [41]. Drawing on the well-established relationship between flat-band potential and band edge positions for different semiconductor types, the E_fb_ of n-type semiconductors lies 0.1–0.3 eV more positive than the E_CB_ at the semiconductor/electrolyte junction, whereas the E_fb_ of p-type semiconductors lies 0.1–0.3 eV more negative than the E_VB_ [41,42]. Applying this empirical correction, the CB positions of LaVO_4_ and Bi_2_O_2_S were estimated to be −0.03 V and −0.86 V vs. NHE, respectively. Complementarily, the optical bandgaps of LaVO_4_ and Bi_2_O_2_S were extracted from Tauc plots constructed from the UV-Vis DRS data (Figure 6b), yielding Eg values of 2.09 eV and 1.20 eV, respectively. Leveraging the fundamental semiconductor energy relation E_CB_ = E_VB_ − Eg [41,42], the remaining band edge positions are further derived: the VB edges of LaVO_4_ and Bi_2_O_2_S were calculated to be +2.06 V and +0.34 V vs. NHE, respectively.

**Figure 13:**
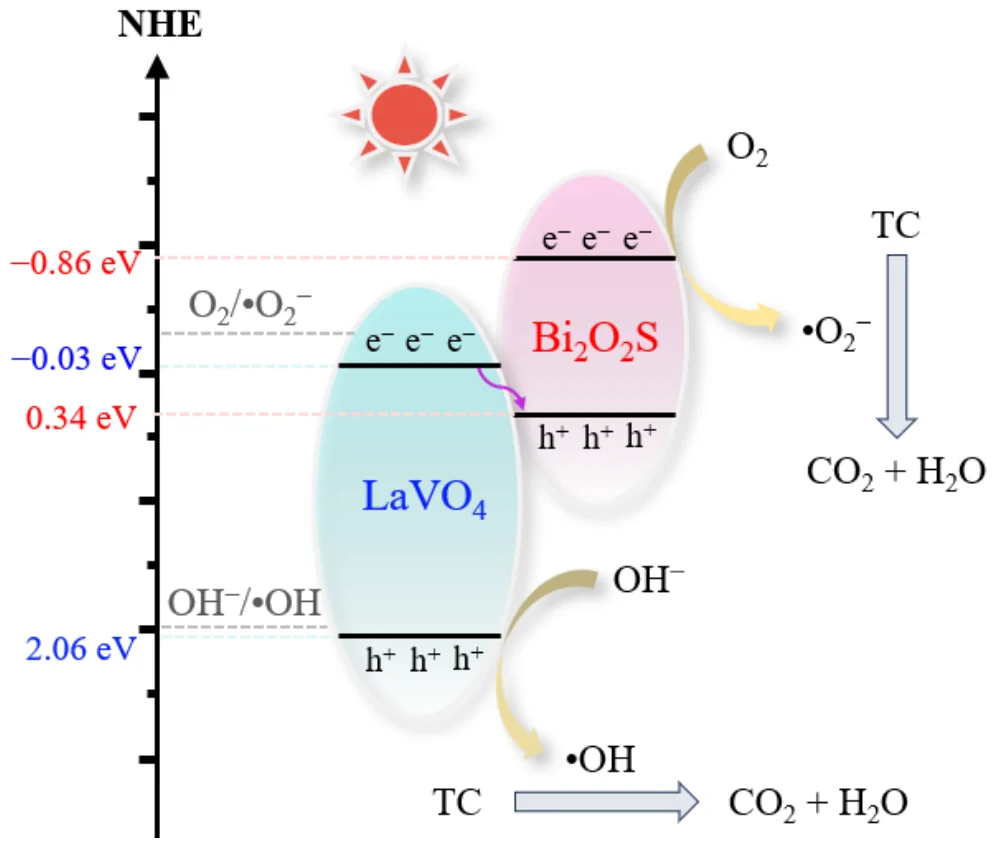
Possible S-scheme heterojunction photocatalytic reaction mechanism for the LaVO_4_/Bi_2_O_2_S heterostructure.

By synthesizing experimental evidence from radical quenching assays, ESR spectroscopy, redox potential thermodynamic assessments, and interfacial charge transfer analysis, a step-scheme (S-scheme) heterojunction photocatalytic mechanism is proposed, as schematically illustrated in Figure 13. Under visible-light irradiation, the LVO/BOS composite absorbs incident photons to generate photogenerated e^−^–h^+^ pairs, which migrate toward the heterojunction interface. A preliminary inference of the II-type heterojunction structure suggests that it would suppress the production of •O_2_^−^ and •OH radicals. As reflected by the band edge positions, the E_CB_ of LaVO_4_ (−0.03 eV vs. NHE) is less negative (i.e., more positive) than the standard redox potential of the O_2_/•O_2_^−^ couple (−0.33 eV vs. NHE), while the E_VB_ of Bi_2_O_2_S (+0.34 eV vs. NHE) is less positive (i.e., more negative) than the standard oxidation potential of the OH^−^/•OH couple (+1.99 eV vs. NHE), rendering the formation of both radicals thermodynamically unfavorable [5,43]. However, the unambiguous detection of both •O_2_^−^ and •OH radicals via ESR spectroscopy and radical trapping experiments confirms their active participation in TC photodegradation, indicating that the type II style fails to account for the altered local reaction environment and surface defect states at the heterojunction interface. To resolve this discrepancy, a refined S-scheme photocatalytic mechanism is proposed, as visualized in Figure 13. In this configuration, the photogenerated electrons in the E_CB_ of LaVO_4_ migrate to the heterojunction interface and recombine with holes in the E_VB_ of Bi_2_O_2_S, effectively suppressing bulk e^−^–h^+^ recombination. Consequently, reducing electrons are retained in the E_CB_ of Bi_2_O_2_S, while oxidizing holes are preserved in the E_VB_ of LaVO_4_. The retained electrons in the E_CB_ of Bi_2_O_2_S (−0.86 eV vs. NHE) possess a sufficiently negative potential to drive the reduction of molecular oxygen to superoxide radicals (•O_2_^−^), as this potential is more negative than the standard redox potential of the O_2_/•O_2_^−^ couple [44,45]. Simultaneously, the holes localized in the E_VB_ of LaVO_4_ (+2.06 eV vs. NHE) exhibit strong oxidative capacity that can oxidize surface hydroxyl groups or water molecules to generate •OH. This S-scheme charge transfer pathway not only maximizes photogenerated e^−^–h^+^ separation efficiency, but also preserves the strongest redox capacities of both charge carriers, leading to enhanced generation of •O_2_^−^ and •OH radicals, which serve as the dominant reactive species driving TC photodegradation.

## 3. Experimental Section

### 3.1. Catalyst Preparation

#### 3.1.1. Preparation of LaVO_4_ Catalyst

The pristine LaVO_4_ was synthesized by adapting the synthetic protocol reported in the prior literature, with only minor modifications to the original procedure [13]. Specifically, stoichiometric quantities of ammonium metavanadate (NH_4_VO_3_, 0.4095 g) and lanthanum nitrate hexahydrate (La(NO_3_)_3_·6H_2_O, 1.5155 g) were precisely weighed and individually dissolved in 35 mL of deionized water under constant magnetic stirring until complete dissolution was achieved. The as-prepared NH_4_VO_3_ solution was first pre-equilibrated in a thermostatic water bath set to 80 °C to stabilize temperature and precursor supersaturation, after which the La(NO_3_)_3_·6H_2_O solution was added dropwise under continuous agitation; the mixture was further stirred for an additional 30 min post-addition to guarantee full homogenization of the precursor sol. The homogeneous mixed sol was then transferred to a 100 mL polytetrafluoroethylene (PTFE)-lined stainless-steel autoclave, hermetically sealed, and subjected to hydrothermal crystallization at 180 °C in an oven for 24 h. Upon completion of the reaction, the autoclave was allowed to cool naturally to ambient temperature, and the obtained precipitate was harvested via centrifugation (the speed is 7546× *g*, i.e., 8000 rpm), followed by three consecutive centrifugal washing cycles with copious deionized water to eliminate surface-adsorbed residual ionic species. Finally, the purified sample was dried in a forced-air drying oven set to 90 °C for 5 h, and subsequently ground using an agate mortar to obtain phase-pure LaVO_4_ powder.

#### 3.1.2. Preparation of Bi_2_O_2_S Catalyst

The single Bi_2_O_2_S was synthesized according to the work of Seling et al. [27]. Specifically, about 1 g of Bi(NO_3_)_3_·5H_2_O was added into 200 mL of deionized water and ultrasonicate for 30 min to obtain a homogeneous, transparent bismuth precursor solution. In a separate vial, 78.53 mg of thiourea (CH_4_N_2_S) and 6.7 mL of hydrazine hydrate (N_2_H_4_·H_2_O) are added and ultrasonicated for 30 min to prepare a mixed sulfur source-reductant solution. The above two solutions are mixed and ultrasonicated for another 30 min to ensure uniform dispersion, after which 1.2 g of KOH and 3.2 g of NaOH are added to the mixed solution. Ultrasonication is continued until the alkali is completely dissolved. The mixture is then stirred at room temperature for 8 h. After the reaction is completed, the generated brown precipitate is collected, washed three times with deionized water via centrifugation to remove impurities, dried in a 150 °C forced-air oven for 12 h, and ground to obtain pure-phase Bi_2_O_2_S powder.

#### 3.1.3. Preparation of LaVO_4_/Bi_2_O_2_S Heterojunction Composites

The composites are prepared via an in-situ compounding method. Following the single-phase Bi_2_O_2_S synthesis procedure up to the step where the alkali is fully dissolved under ultrasonication, predetermined masses of pure-phase LaVO_4_ powder are added to the mixed solution to achieve theoretical mass ratios of 1:1, and 1:2 (LaVO_4_:Bi_2_O_2_S), labeled as LVO/BOS and LVO/2BOS, respectively. The mixture is then stirred continuously at room temperature for 12 h to ensure full hybridization of the heterojunction structure. After the reaction, the precipitate is collected, washed three times with deionized water via centrifugation to remove surface-adsorbed residual reactants, and finally dried in a 150 °C forced-air oven for 12 h to obtain LaVO_4_/Bi_2_O_2_S heterojunction composites with different loading ratios.

### 3.2. Catalyst Characterization

The crystallographic architecture of the catalysts was deciphered via high-resolution XRD analysis employing a Bruker D8-Advance diffractometer utilizing Cu Kα radiation. S_BET_ and hierarchical pore size distribution were precisely quantified at 77 K through N_2_ physisorption isotherms acquired on a Micromeritics (Norcross, GA, USA) ASAP 2020 adsorption–desorption system. Before the tests, the samples were pre-treated under vacuum at 300 °C for 8 h to ensure complete drying and degassing. Surface chemistry and molecular bonding configurations were comprehensively interrogated using FT-IR (FTIR-8400S, Shimadzu, Kyoto, Japan) and monochromatic Al Kα XPS (ESCALAB 250, Thermo Fisher Scientific, Waltham, MA, USA), respectively. Optical bandgap profiles and sub-bandgap absorption dynamics were probed via UV-Vis DRS across 200–1200 nm, with BaSO_4_ serving as the inert reflectance standard (UV3600IPLUS, Shimadzu, Kyoto, Japan). PL spectra were recorded at ambient temperature using a HITACHI F-7000 spectrophotometer (F-7000, Hitachi, Tokyo, Japan) to assess charge carrier recombination kinetics. Prior to each measurement, approximately 5 mg of the sample is weighed and dispersed into 10 mL of anhydrous ethanol, followed by ultrasonication for about 10 min. Nanoscale morphology and atomic-scale structural motifs were correlated through FE-SEM (FE-SEM, SU8600, Hitachi, Tokyo, Japan) and TEM (FEI Talos F200X, FEI, Hillsboro, OR, USA). Critically, transient radical intermediates were detected and quantified via ESR spectroscopy (JES-FA300, Bruker, Karlsruhe, Germany), employing 5,5-dimethyl-1-pyrroline N-oxide (DMPO) as the spin-trapping agent to selectively capture hydroxyl (•OH) and superoxide (•O_2_^−^) radicals through stable spin-adduct formation.

### 3.3. Photoelectrochemical Measurements

All electrochemical characterizations were conducted on a CHI 660D potentiostatic workstation (Chenhua, Shanghai, China). A standard three-electrode electrochemical cell was assembled for all tests, wherein a platinum (Pt) wire functioned as the auxiliary (counter) electrode, Ag/AgCl electrode served as the reference electrode, and the as-fabricated catalyst films were deployed as the working electrodes. Photocurrent density-response measurements were acquired in a quiescent 0.5 M Na_2_SO_4_ aqueous electrolyte under simulated solar irradiation delivered by a 300 W xenon (Xe) arc lamp. Electrochemical impedance spectroscopy (EIS) measurements were performed in the identical Na_2_SO_4_ electrolyte, with a 10 mV amplitude alternating current (AC) small-signal perturbation applied across a swept frequency range of 10^5^ to 10^−1^ Hz, to interrogate the interfacial charge transfer kinetics and charge transport resistance of the electrode/electrolyte interface.

### 3.4. Photocatalytic Tests

The visible-light-driven photocatalytic degradation performance of the as-synthesized photocatalysts was systematically appraised and benchmarked using TC as a representative refractory organic model contaminant. A 300 W Xe arc lamp (model: PLS-SXE 300BF, Merry Change, Beijing, China) fitted with a 420 nm UV-cutoff filter was deployed as the simulated visible-light irradiation source. For performance evaluation, 50 mg of the as-synthesized photocatalyst was homogeneously dispersed in 50 mL of 10 mg L^−1^ tetracycline aqueous solution, followed by 60 min of continuous stirring in absolute darkness to preclude photonic interference during the pre-equilibration stage and ensure complete establishment of adsorption–desorption equilibrium at the catalyst-solution interface. At predefined temporal intervals (every 15 min), 3 mL reaction aliquots were promptly withdrawn from the suspension, rapidly filtered through 0.22 μm microfiltration membranes, and subsequently quantified via ultraviolet-visible (UV-Vis) absorption spectroscopy at the characteristic λ_max_ of 357 nm for TC. The photocatalytic degradation reaction was conducted under visible-light irradiation over a total duration of ~90 min. Additionally, the operational durability and long-term structural stability of the as-prepared LVO/BOS heterojunction photocatalyst were systematically probed via five sequential cyclic degradation runs, during which the photocatalyst was retrieved, reactivated, and redeployed following each individual cycle to evaluate its recyclability and quantify the extent of photocatalytic activity retention across successive cycles. The specific steps are as follows: after each photocatalytic reaction, the catalyst is collected from the reaction solution via centrifugation at 8000 rpm (corresponding to 7546× *g*) for 10 min. The collected catalyst is then dried at 60 °C for 24 h. The mass of catalyst lost during each operation is replenished with fresh catalyst to ensure that the total catalyst loading for each run is 50 mg.

## 4. Conclusions

In conclusion, a binary S-scheme LaVO_4_/Bi_2_O_2_S heterojunction has been successfully constructed via a simple hydrothermal strategy. Comprehensive physicochemical characterization coupled with photocatalytic activity assays reveal that the incorporation of Bi_2_O_2_S not only streamlines interfacial charge transport across the LaVO_4_ matrix, but also endows the composite with markedly enhanced surface catalytic oxidative potency. Under visible-light-driven photocatalytic TC degradation, the optimized LVO/BOS heterojunction achieves a degradation efficiency of 89%, far outperforming its pristine single-component counterparts (bare LaVO_4_: 41%; monolithic Bi_2_O_2_S: 44%). The intrinsic S-scheme charge transfer pathway drives efficient spatial charge separation, wherein photogenerated electrons are preferentially populated on the negative conduction band of Bi_2_O_2_S, while holes migrate to the positive valence band of LaVO_4_, thereby amplifying the redox capability of the separated charge carriers. Collectively, this work provides novel insights into the rational design of visible-light-responsive high-efficiency photocatalysts, and paves the way for the scalable solar-energy-driven remediation of organic pollutants.

## Figures and Tables

**Table 1:** The fitting parameter of EIS results of all the samples in Figure 8b.

Sample	Rs (ohm)	Rct (ohm)	Chi-Squared
LaVO_4_	14.53	53.11	0.0001145
Bi_2_O_2_S	15.63	44.39	0.0017792
LVO/BOS	14.91	21.7	0.0013495
LVO/2BOS	15.44	35.99	0.0011116

## Data Availability

The data presented in this article can be obtained from the corresponding author upon reasonable request.
